# Developing Malawi's Universal Health Coverage Index

**DOI:** 10.3389/frhs.2021.786186

**Published:** 2022-02-10

**Authors:** Martina Mchenga, Gerald Manthalu, Atamandike Chingwanda, Emily Chirwa

**Affiliations:** Ministry of Health, Lilongwe, Malawi

**Keywords:** Universal Health Coverage (UHC), service coverage, financial risk protection, health system, out-of-pocket (OOP) payments, catastrophic health expenditure (CHE)

## Abstract

The inclusion of Universal Health Coverage (UHC) in the Sustainable Development Goals (target 3.8) cemented its position as a key global health priority and highlighted the need to measure it, and to track progress over time. In this study, we aimed to develop a summary measure of UHC for Malawi which will act as a baseline for tracking UHC index between 2020 and 2030. We developed a summary index for UHC by computing the geometric mean of indicators for the two dimensions of UHC; service coverage (SC) and financial risk protection (FRP). The indicators included for both the SC and FRP were based on the Government of Malawi's essential health package (EHP) and data availability. The SC indicator was computed as the geometric mean of preventive and treatment indicators, whereas the FRP indicator was computed as a geometric mean of the incidence of catastrophic healthcare expenditure, and the impoverishing effect of healthcare payments indicators. Data were obtained from various sources including the 2015/2016 Malawi Demographic and Health Survey (MDHS); the 2016/2017 fourth integrated household survey (IHS4); 2018/2019 Malawi Harmonized Health Facility Assessment (HHFA); the MoH HIV and TB data, and the WHO. We also conducted various combinations of input indicators and weights as part of sensitivity analysis to validate the results. The overall summary measure of UHC index was 69.68% after adjusting for inequality and unadjusted measure was 75.03%. As regards the two UHC components, the inequality adjusted summary indicator for SC was estimated to be 51.59% and unadjusted measure was 57.77%, whereas the inequality adjusted summary indicator for FRP was 94.10% and unweighted 97.45%. Overall, with the UHC index of 69.68%, Malawi is doing relatively well in comparison to other low income countries, however, significant gaps and inequalities still exist in Malawi's quest to achieve UHC especially in the SC indicators. It is imperative that targeted health financing and other health sector reforms are made to achieve this goal. Such reforms should be focused on both SC and FRP rather than on only either, of the dimensions of UHC.

## Introduction

Universal health coverage (UHC) inclusion in the sustainable development goal (SDG) targets (goal target 3.8), and the national commitments to achieve it, has emphasized the need to measure it and track its progress over time (1, 2). According to the World Health Organization (3), the goal of UHC in general is to ensure that everyone irrespective of their socio-demographic characteristics has access to quality healthcare services when needed without the risk of going bankrupt. Maeda et al. (4) argued that attaining UHC can lead to positive health outcomes, thus helping develop human capital. Development of human capital can then promote job creation, increase financial protection and reduce poverty, promote economic inclusion, and strengthen health security and, thus, macro-stability (4).

According to Wagstaff et al. (2), UHC is defined as having two dimensions: (1) essential health services coverage (3.8.1), which is defined as everyone, irrespective of ability to pay, getting the services they need and (2) financial protection (3.8.2), defined as nobody suffering financial hardship as a result of receiving needed care. The first captures population service coverage, whereas the latter captures the financial expenditures by population group. The summary measure for UHC, therefore, is a combination of the two dimensions (2, 3).

The way UHC is monitored, however, varies by country. While some countries use tracer indicators covering both the service coverage (SC) and financial risk protection (FRP) dimensions (5, 6), other countries have developed composite indices that capture both dimensions (7, 8). Most of this work has been inspired by the pioneering work of the WHO and the World Bank (WB) (9). Malawi is a member of the United Nations SDG and is committed to achieving UHC by 2030 (10). However, the country does not have either a set of agreed tracer indicators or a composite index for monitoring UHC. Given the current policy directions to attain UHC by 2030, the measurement and the tracking of UHC are even more vital.

The goal of this paper, therefore, was to develop a UHC index for Malawi and provide a baseline on which progress toward the attainment of UHC can be measured. To do this, we adopted the WHO and the WB framework for monitoring UHC and the proposal by Wagstaff et al. (2) and Barasa et al. (7) on how to implement the framework. The most recent data available were used for the analysis. Findings from this analysis will inform policy in identifying priority areas for improvement to fast-track the progress toward the attainment of UHC in Malawi.

### Healthcare Services Delivery in Malawi

Healthcare services in Malawi are primarily provided by the public sector, which provides the majority (52%) of the health services (11). There are four service delivery levels of care, namely, community, primary, secondary, and tertiary, with inter-level referrals as required (10). At the *community level*, health services are provided by health surveillance assistants (HSAs), health posts, dispensaries, village clinics, and maternity clinics (10). Under the Ministry of Health (MoH)-established integrated community case management (iCCM) approach, HSAs are trained and deployed in hard-to-reach areas to provide both promotive and preventive health services for uncomplicated cases of malaria, pneumonia, diarrhea, newborn sepsis, and malnutrition. Approximately 8,900 HSAs have been trained and deployed in all of Malawi's 29 districts to provide iCCM services, representing 94% of the total hard-to-reach areas identified nationwide (10).

The second level is the *primary healthcare*, which consists of smaller-level facilities such as health centers and community and rural hospitals (10). The primary healthcare level delivers both inpatient and outpatient services, and services are mainly provided by midwife assistants, nursing assistants, and clinicians. The *secondary level* constitutes district hospitals, which offer both inpatient and outpatient services to the local catchment population and function as referral facilities for primary healthcare facilities. The services at the district level are provided by nurses, midwives, and general practitioners. Lastly, the *tertiary level* of services comprises five central hospitals, each of which provides specialized health services. At this level of care, you will find specialists in different areas such as neurosurgeons and gynecologists, among others.

Besides the government/public sector, other actors include private for-profit (PFP) and private not-for-profit (PNFP). PNFP includes non-governmental organizations (NGOs), company clinics, Christian health association (Christian Health Association of Malawi, CHAM) facilities, and other faith-based owned facilities. PNFP is the second (25%) largest provider of care from the government and mainly provides secondary level of care, followed by a large number of independent private health facilities that provide the remaining 23% of the services (11).

### UHC-Inspired Reforms in Malawi

The government of Malawi is committed to providing accessible and affordable healthcare to all its citizens. To meet the health needs of its population, the government developed an essential healthcare package (EHP) consisting of a set of interventions that provide the best value for money (11). First introduced in 2004, EHP interventions are provided free of charge at primary healthcare levels in all public health facilities (11). Over the years, the EHP has been revised in line with the medium-term Health Sector Strategic Plan (HSSP): 2011–2016 HSSP (12) and 2017–2022 HSSP II (10). In each case, the EHP included interventions that are cost-effective and deal with major causes of morbidity and mortality in Malawi, with a focus on ensuring equity (13). As described in the HSSP II (10) and as shown in Table 1, the range of services in the package include reproductive, maternal, neonatal, and child healthcare (RMNCH) services, vaccinations, malaria, nutrition, HIV and AIDS, and tuberculosis, among others (11).

**Table 1:** Interventions included in the essential health package (EHP).

**EHP category**	**Intervention package**
RMNCH	ANC package including tetanus toxoid, deworming, iron and folic supplements, syphilis detection, IPT, insecticide-treated bed net (ITN) distribution to pregnant women, and urinalysis
	Modern family planning including all modern contraceptive methods
	Delivery package
Vaccine-preventable diseases	Essential vaccine package including rotavirus vaccine, measles, polio, and HPV, among others
Malaria	First-line uncomplicated malaria
	Complicated malaria treatment
	Malaria diagnosis
Integrated management of childhood illnesses	ARIs
	Diarrheal diseases
	Nutrition
	Malaria diagnosis
Community health package	Community health package
NTDs	Treatment and mass drug administration
HIV/AIDS	HIV prevention
	HIV testing
	HIV treatment
Nutrition	Vitamin A supplementation
	Deworming
	Management of severe malnutrition
TB	TB testing
	TB treatment
NCDs	Treatment of injuries
	Mental health treatment
	Testing of pre-cancerous cells
	Diabetes and hypertension treatment
Oral health	Tooth pain treatment

*MoH (11)*.

*ANC, antenatal care; IPT, intermittent presumptive treatment; RMNCH, reproductive, maternal, neonatal and child healthcare; HPV, human papillomavirus; ARIs, acute respiratory infections; NTDs, neglected tropical diseases; TB, tuberculosis; NCDs, non-communicable diseases*.

However, the network of public health services does not, by the government's definition of physical access (within an 8-km radius of a public health facility), cater to 100% of the population, especially in rural areas where the need for affordable health services is the greatest. Despite the increase in the proportion of the population that resides within an 8-km radius of a health facility (health centers and hospitals), from 81% in 2011 to 90% in 2016 (14), there is still a proportion of the population that remains underserved, especially in rural and hard-to-reach areas.

Although the primary provider of healthcare in Malawi is the government, the CHAM also plays a significant role in the provision of healthcare services in Malawi. About 75% of health services in remote and rural areas are provided by CHAM facilities (15). Currently, an estimated 3.7 million Malawians live in CHAM catchment areas. Since 2006, as a way of increasing equitable healthcare access to basic healthcare services among the rural poor, the government started exploring strategic partnerships with CHAM through an arrangement called service legal agreement (16). In essence, CHAM would provide health services free of charge to the user and the government would reimburse CHAM for the cost of providing the health services.

## Health Financing

There are three main financing actors in Malawi's health sector: government, donor, and private. The government finances the health sector using public funds from tax collection, return on government assets, and other sources. Donors, on the other hand, finance the sector through supporting the government's development budget, procuring medical commodities, and directly supporting programs and other providers (14). Last but not least, private financing comes from household out-of-pocket (OOP) expenditure, firms, and private insurance providers. The key indicators for health financing between 2015 and 2018 are described in Table 2.

**Table 2:** Key health accounts findings.

**Indicators**	**2015/2016**	**2016/2017**	**2017/20118**	**Average**
Total health expenditure (THE) (MWK million)	429,095	495,353	502,773	476,694
THE (US $ million)	678.95	685.14	693.48	687.17
Per capita total expenditure on health (at average US $ exchange rate)	40.3	39.4	39.5	39.8
THE as a percentage of GDP	11.3	10.7	9.8	10.6
Government expenditure on health as a percentage of THE	24.7	22.7	24.4	23.9
Donor expenditure on health as a percentage of THE	58.4	59.9	57.6	58.6
Total private expenditure as a percentage of THE	16.9	17.5	17.9	17.4
Out-of-pocket expenditure on health as a percentage of private expenditure on health	73.5	72.7	70.8	72.3
Out-of-pocket expenditure on health as a percentage of THE	12.4	12.7	12.7	12.6
Health insurance as a percentage of private expenditure on health	26.5	27.3	29.2	27.7
Health insurance corporations as a percentage of THE	2.8	3.8	4.3	3.6

*MoH (14)*.

*GDP, gross domestic product*.

Several issues are apparent from Table 2. (1) The Malawian health system faces many challenges to effectively provide primary healthcare services, among them being inadequate funding. For example, in recent years, the budget allocation of this sector has been consistently lower than the recommended Abuja target of 15% of the gross domestic product (GDP). (2) The health sector is heavily dependent on donor funding. (3) Household OOP expenditures are the main contributors to private health financing.

Despite the provision of free primary healthcare services in all public facilities and the removal of user fees in selected CHAM facilities (16), Malawi still lags behind in attaining the UHC goals of providing quality healthcare services and ensuring FRP. For instance, a study conducted by Leslie et al. (17) reported that, for key maternal and child health interventions in Malawi, effective coverage is 24.7%. A study conducted by Mchenga et al. (18) in 2017 reported that the incidence of catastrophic expenditure in Malawi varied between 0.73 and 9.37%. The researchers used thresholds of 10–40% of non-food expenditure, leading to an addition of 0.93% of the population being pushed into poverty due to OOP expenditures.

Currently, voluntary private medical insurance plays a negligible role in financing healthcare in Malawi. For example, between 2015 and 2018, private health insurance contributed an average of 3.6% to total health spending, as shown in Table 2. Generally, pooling in Malawi is significantly fragmented and has a limited redistributive capacity. Estimates of the resource mapping study suggested that only 26% of all health resources are in any form of pool (14). Furthermore, the donor landscape is highly segmented, with most donors channeling their support “off-budget” outside of government systems (14). The challenge with off-budget funding is that it is difficult for the government to have proper oversight in the allocation of resources and implementation of programs. This results in misalignment with government priorities, high transaction costs, and inefficient resource allocation, which tends to exacerbate inequities in the targeting and delivery of health programs (14). There is also little joint planning between the major development partners and the Ministry of Health and Population (MoHP) and district health management teams.

## Methods and Data

### Study Framework

To compute the UHC index, we adopted the framework proposed by Wagstaff et al. (2). The UHC index therefore is a representation of two components: SC and FRP. Based on the framework, the SC component is made up of two domains capturing essential health interventions: prevention and treatment indicators. Similarly, the FRP dimension also comprises two domain indicators: incidence of catastrophic healthcare spending and the proportion of the population that is impoverished by OOP. Additionally, given that at the core of UHC is equity, it is essential that the tracking of UHC progress takes into account an equity analysis. This is done by computing an achievement index that relates directly to the concentration index (2). The concentration index is an important tool for measuring inequalities, and it quantifies the degree of socioeconomic-related inequality in a given health variable (2). How the achievement index is computed for each of the indicators is explained in the proceeding sections.

### Summary of the UHC Index

As previously indicated, we used the approach by Wagstaff et al. (2) to summarize the UHC index, which uses geometric means. The argument given for the use of geometric means is that they are more sensitive to extreme values. As a result, they implicitly assign more weight to health services with lower coverage and are less sensitive to the scale on which input variables are measured compared to when using arithmetic means (9).

Using the two dimensions SC and FRP, the UHC index is presented as the geometric mean as follows:

(1) $$\begin{array}{r} \text{UHC }=\text{ S}{\text{C}}^{\varphi }\cdot \text{F}{\text{P}}^{1-\varphi } \end{array}$$

where SC is the service coverage, φ is a weight for SC, FRP is the financial risk protection, and 1 − φ is the weight for FRP. In terms of the exact weights to be used, most policy makers use 0.5 for each (2). In this study, we adopted the same weights. The range of the index is between 0 and 100, with higher numbers representing higher UHC progress.

The individual components are in turn calculated as follows. The SC is calculated as a geometric mean of two dimensions: prevention and treatment. Firstly, the prevention dimension was defined as follows:

(2) $$\begin{array}{r} \text{S}{\text{C}}_{\text{P}}=\text{S}{\text{C}}_{{\text{P}}_{1}}^{\alpha_{1}}\text{S}{\text{C}}_{{\text{P}}_{2}}^{\alpha_{2}}\ldots \text{S}{\text{C}}_{{\text{P}}_{\text{n}}}^{\alpha_{\text{n}}} \end{array}$$

where ${\text{SC}}_{P_{1}}^{\alpha_{1}}$ is prevention indicator 1 with equal weights of α_*i*_, *i* = 1 … *n*. Secondly, the treatment dimension was defined as follows:

(3) $$\begin{array}{r} \text{S}{\text{C}}_{\text{T}}=\text{S}{\text{C}}_{{\text{T}}_{1}}^{\beta_{1}}\text{S}{\text{C}}_{{\text{T}}_{2}}^{\beta_{2}}\;\ldots \text{ S}{\text{C}}_{{\text{T}}_{\text{n}}}^{\beta_{\text{n}}} \end{array}$$

where ${\text{SC}}_{T_{1}}^{\beta_{1}}$ is treatment indicator 1 with weight β_*i*_, *i* = 1 … *n*. In line with Wagstaff et al. (2) and Barasa et al. (7), the geometrical mean of the treatment indicators was computed assigning the indicator for hospital admission a weight of 50% and the other 50% being shared equally among the rest of the treatment indictors.

With both components calculated, the summary measure for SC was then calculated as follows:

(4) $$\begin{array}{r} \text{SC}=\text{S}{\text{C}}_{\text{P}}^{\pi }.\text{S}{\text{C}}_{\text{T}}^{1-\pi } \end{array}$$

where ${\text{SC}}_{P}^{\pi }$ is the prevention dimension with weight π and ${\text{SC}}_{T}^{1-\pi }$ is the treatment dimension with weight 1 − π. Wagstaff et al. (2) assigned the prevention indicator a lower weight of 25%, which is relatively higher than the share of prevention in total health spending in the OECD (Organization for Economic Co-operation and Development) countries and in Asia. In this study, however, we assigned a 30% weight to the prevention indicator based on the findings of the 2020 National Health Accounts, which showed that, between 2015/2016 and 2017/2018, the health spending on preventive care as a share of the total health spending was an average of 30.8% (14). Nevertheless, as part of the sensitivity analysis, we also used 25% to assess whether there would be a difference, but we did not observe any difference in the summary measure of SC.

We calculated the population-level averages for each of the SC indicators to obtain population-level estimates. To account for inequality in service coverage, concentration indices for each of the SC indicators were computed ranked by inequality components such as household income level and education level of the household head, among others, depending on the data. We then computed an achievement index to account for the differences across demographic groups. This was done by assigning an achievement score below the population mean to variables with high SC rates and were pro-rich and *vice versa* (2). Put simply, this was done by computing the multiplication of the population mean of the SC indicator by its concentration index complement (2, 19), such that each individual intervention and composite intervention was adjusted for inequality, giving us an inequality-adjusted SC summary measure.

Like the SC component, the financial risk protection component, *FRP*, is a geometric mean of two domains. The first is the incidence of “catastrophic” health payments, FRP_CATA_. We defined catastrophic health payments as payments that exceeded the 40% threshold of non-food expenditures, as proposed by the WHO. The 40% threshold was chosen because it takes into account the money that remains after all the basic needs have been met. As such, it is the best estimate of capacity to pay for healthcare expenditure (7). We then computed the complement of the incidence of catastrophic health expenditures (CHEs) in order to obtain the proportions of households that did not suffer from any CHEs (2). The proportions of households with no CHEs were considered to have some level of FRP. This was defined as follows:

(5) $$\begin{array}{r} \text{FR}{\text{P}}_{\text{CATA}}\;=\;\frac{\left(1\;-\text{ Cata})\;(1\;-\text{ Cat}{\text{a}}_{\text{MAX}}\right)}{\left(1\;-\;{\text{Cata}}_{\text{MIN}}\right)\;\left(1\;-\;{\text{Cata}}_{\text{MAX}}\right)} \end{array}$$

where Cata is the proportion of households incurring catastrophic expenditures; the subscripts MIN and MAX represent minimum and maximum values, respectively. To examine inequality in FRP, we computed the population means of the complements of CHE indicators and their concentration indices. To account for the differences in the distribution of catastrophic spending across demographic groups, we multiplied the indicator with its corresponding achievement index.

The other component of FRP is the “impoverishing” payments, FRP_IMPOV_. This indicator considers individuals who were pushed into poverty by OOP spending over a given period (usually a 1-year period). The complement of which is the proportion of individuals who do not get into poverty due to OOP expenditures and was calculated as follows:

(6) $$\begin{array}{r} \text{FR}{\text{P}}_{\text{IMPOV}}\;=\;\frac{\left(1\;-\text{ Impov}\right)\;\left(1\;-\text{Impo}{\text{v}}_{\text{MAX}}\right)}{\left(1\;-{\text{Impov}}_{\text{MIN}}\right)\;\left(1\;-{\text{Impov}}_{\text{MAX}}\right)} \end{array}$$

However, on slight departure to Wagstaff et al. (2), Barasa et al. (7) defined the impoverishment indicator as the percentage of the population that is poor and had reported to spending OOP for health (i.e., the proportion of the poor population that has gotten deeper into poverty) plus the percentage of non-poor individuals who got poor due to OOP spending. The authors' justification is that, in the context of UHC, poor people are already in a vulnerable financial state as such FRP means that these people should not spend money OOP to access health services. Given that the majority of the population in Malawi is poor, just like in most low- and middle-income countries (LMICs), we adopted the definition of Barasa et al. (7) of impoverishment to avoid overstating FRP. Therefore, the complement of the FRP indicator was computed as the percentage of the population that does not get poor or is deep into poverty by spending OOP for health.

### Validation/Sensitivity Analysis of the UHC Index

To validate our results, we performed the following sensitivity analyses: (1) we computed the inequality-unadjusted geometric summary of the UHC index. (2) While the starting point of the analysis the assumption made was that treatment interventions had a 70% weight in comparison to prevention interventions in computing the SC summary measure, equal weights of 50% each for the prevention and treatment indicators were used in the sensitivity analysis. (3) For hospital admissions, the base case scenario assumed a 50% in comparison to the other treatment interventions; the sensitivity analysis assigned equal weights to all treatment interventions. (4) Lastly, a reduced summary measure of UHC was computed with some of the service capacity indicators excluded.

### Selection of Indicators and the Guiding Principles

In selecting indicators, the WHO and the WB advised that authors should make sure that they are relevant, of high quality, and that data are available (9). Based on these guidelines, we therefore selected indicators based on the following principles: firstly, the index captures both prevention and treatment indicators. Prevention indicators include both health promotion and prevention of illnesses, whereas treatment indicators include remedial, rehabilitation, and palliation services (1). For policy relevance, the analysis in this study was based on indicators capturing essential services that were included in the Malawi health benefit package, as described in Table 1 (10). Secondly, the index covered key RMNCH services and communicable and non-communicable diseases. Lastly, due to data limitations, only indicators whose data are routinely available through routine health surveys were included. The indicators included in this analysis are discussed below.

### Service Coverage Indicators

As explained earlier, SC indicators comprised two domain indicators: prevention and treatment indicators. Between the two SC components, the easier one to operationalize was the prevention component, with data easily available. To capture the prevention component, nine indicators were used: whether a pregnant woman had received at least four antenatal care (ANC) visits; whether a child was fully immunized; cervical cancer screening; whether a pregnant woman was given iron–folic acid (IFA); at least two tetanus toxoid (TT) injections during pregnancy; use of mosquito net for children under 5; whether a pregnant woman used a mosquito net; proportion of women on modern contraceptive methods; and met the needs for family planning.

On the other hand, due to data scarcity, the treatment component was difficult to operationalize (2). Nonetheless, 13 indicators were identified and used for the analysis: skilled birth attendant (SBA); treatment of diarrhea in children with oral rehydration salts (ORS) or a homemade solution; whether a child with acute respiratory infection (ARI) got medical treatment; mother or child postnatal check by skilled personnel; effective tuberculosis (TB) treatment coverage; proportion of HIV-positive people receiving antiretroviral therapy (ART); treatment coverage among people with cardiovascular diseases; proportion of people with diabetes receiving treatment; and whether the baby was delivered in a health facility.

Under the treatment domain, as recommended by the WHO and WB (9), service capacity and access indicators were accounted for to capture the capability of the Malawian health system to adequately provide healthcare access. This includes key areas such as everyday medical examinations, mental illness treatment, emergency care, and surgical procedures (9). The indicators chosen to proxy service capacity and access included hospital bed density (proxies hospital admission), core health professional distribution and density, which include general medical doctors, specialist medical doctors, non-physician clinicians, nursing professionals, and midwifery professionals, access to essential medicines, and health security measure (9).

In summary, the chosen service coverage indicators met three requirements: (1) they included an element of prevention and treatment; (2) the indicators covered services capturing the healthcare needs of individuals during the entire life span, i.e., children, reproductive age population, and the elderly; and (3) the health service indicators represented a variety of needs, such as child and maternal healthcare, treatment for infectious and non-communicable diseases, and facility-level outpatient and inpatient care.

Detailed descriptions of the service indicators are given in Table 3. It should be noted, however, that the definition for each indicator was adopted from the WHO and WB report that tracks UHC in selected LMICs (9).

**Table 3:** Operational definitions for indicators of the universal health coverage (UHC) index in Malawi.

**Dimension**	**Coverage indicators**	**Definition**	**Numerator**	**Denominator**	**Data source**	**Data quality**
Prevention indicators	Iron and folic acid (≥100)	Percentage of pregnant women who took iron and folic acid supplementation	Number of women who took iron and folic acid tablets during pregnancy	Total number of women who delivered in the last 2 years	DHS/MICS	Uses sampling weights to ensure quality
	TT (2 injections)	Percentage of pregnant women immunized with at least two TT injections	Number of women immunized with at least 2 TT injections during pregnancy	Total number of women who delivered in the last 2 years	DHS	Uses sampling weights to ensure quality
	≥ 4 Antenatal checkups	Percentage of pregnant women with at least 4 ANC checkups during pregnancy	Number of pregnant women with at least 4 ANC visits	Total number of women aged 15–49 years who delivered in the last 2 years	DHS	Uses sampling weights to ensure quality
	Contraceptive prevalence rate	Percentage of couples using any method of contraception	Number of couples using any method of contraception	Total number of eligible couples	DHS/MICS	Uses sampling weights to ensure quality
	Full immunization	Percentage of 1-year-old children who are fully immunized	Number of 1-year-old children who have received 3 doses of a vaccine containing diphtheria, tetanus, and pertussis; 1 dose of BCG; 3 doses of polio; and 1 dose of measles vaccine	Total number of 1-year-old children	MICS	Uses sampling weights to ensure quality
	ORS use rate	Percentage of children under 5 who received ORS for diarrhea episode in the last 2 weeks	Number of children under 5 given ORS for diarrhea in the last 2 weeks	Total number of children under 5 having episodes of diarrhea in the last 2 weeks	MICS	Uses sampling weights to ensure quality
	ITN coverage for malaria prevention	Percentage of population who slept under an ITN the previous night.	Number of people who slept under an ITN	Total population	DHS/MICS	Uses sampling weights to ensure quality
	Improved water and sanitation	Percentage of households using improved water and improved sanitation facilities	Population living in a household with drinking water from: piped water into dwelling, plot, or yard; public tap/stand pipe; tube well/borehole; protected dug well; protected spring; or rainwater collection AND living in a household with: flush or pour-flush to piped sewer system, septic tank, or pit latrine; ventilated improved pit latrine; pit latrine with slab; or composting toilet	Total population	DHS	Uses sampling weights to ensure quality
	Tobacco control	Age-standardized prevalence of adults ≥15 years not smoking tobacco in the last 30 days	Adults 15 years and older who have not smoked tobacco in the last 30 days	Adults 15 years and older	STEP Survey	Uses a standardized tool developed by the WHO
	Cervical cancer screening	Cervical cancer screening among women aged 30–49 years (%)	Women ages 30–49 who have tested for cervical cancer	Total number of women aged 30–49 years in the sample	STEPs Survey	Uses a standardized tool developed by the WHO
Treatment indicators	Treatment of cardiovascular disease	Age-standardized prevalence of raised blood pressure among adults aged 18+	Number of adults aged 18 or older with systolic blood pressure ≥140 mmHg or diastolic blood pressure ≥90 mmHg	Number of adults aged 18 or older	STEPS Survey	Uses a standardized tool developed by the WHO
	Management of diabetes	Age-standardized prevalence of raised blood glucose among adults 18+	Number of adults aged 18 or older on medication for raised blood glucose	Number of adults aged 18 or older	STEPS Survey	Uses a standardized tool developed by the WHO
	Institutional delivery	Percentage of pregnant women delivering in any level of public or private institution	Number of women delivering in any level of public or private institution	Total number of women who delivered in the last 2 years	DHS	Uses sampling weights to ensure quality
	Postnatal care	Percentage of women who reported having received postnatal checkups within 6 weeks after delivery	Number of women who received postnatal check within 6 weeks after delivery	Total number of live births	DHS	Uses sampling weights to ensure quality
	Medical treatment for acute respiratory infection (ARI)/pneumonia in children	Percentage of children under 5 with suspected pneumonia (cough and difficulty breathing NOT due to a problem in the chest and a blocked nose) in the 2 weeks preceding the survey taken to an appropriate health facility or provider	Number of children under 5 with suspected pneumonia in the 2 weeks preceding the survey taken to an appropriate health provider	Number of children under 5 with suspected pneumonia in the 2 weeks preceding the survey	MICS/DHS	Uses sampling weights to ensure quality
	Tuberculosis treatment	Percentage of incidence TB cases that are detected and successfully treated in a given year	Number of new and relapse cases detected in a given year and successfully treated	Number of new and relapse cases in the same year	MoH NTP annual report	Routinely conducted by the MoH and cross-checked for any errors
	HIV treatment	Percentage of people living with HIV currently receiving antiretroviral therapy (ART)	Number of adults and children who are currently receiving ART at the end of the reporting period	Number of adults and children living with HIV during the same period	MoH HIV data	Routinely conducted by the MoH and cross-checked for any errors
	Hospital^a^ access	Hospital (inpatient) admissions per population per year	Number of inpatient admissions (or discharges) per year	Total population	Health facility census data	Data collected by trained health workers at the MoH
	Health worker density	Health professionals (physicians, psychiatrists, surgeons, nurses, and midwives) per capita, relative to the maximum thresholds for each cadre	[[Inline Image]]Number of physicians, psychiatrists, surgeons, nurses, and midwives	Total population	Facility census data Population census	Data collected by trained health workers at the MoH
	Access to essential medicines	Percentage of health facilities with essential tracer medicines	Number of facilities with essential tracer medicines in stock	Total number of health facilities	Facility census data	
	Health security	International Health Regulations (IHR) core capacity index, which is the average percentage of attributes of 13 core capacities that have been attained at a specific point in time. The 13 core capacities are: (1) national legislation, policy, and financing; (2) coordination and national focal Point communications; (3) surveillance; (4) response; (5) preparedness; (6) risk communication; (7) human resources; (8) laboratory; (9) points of entry; (10) zoonotic events; (11) food safety; (12) chemical events; and (13) radio nuclear emergencies.	Number of attributes attained	Total number of attributes	WHO data	

*TT, tetanus toxoid; ANC, antenatal care; TB, tuberculosis; BCG, Bacillus Calmette–Guérin vaccine; ORS, oral rehydration salts; ITN, insecticide-treated bed net; DHS, demographic and health survey; MICS, multiple indicator cluster survey; MoH, ministry of health; STEPS, stepwise approach for NCD risk factor surveillance*.

a Hospital access was calculated based on inpatient bed density and adopted from the WHO/WB definition (9). The bed density values were rescaled and capped based on the threshold values defined in the harmonized health facility survey. Inpatient bed density had a threshold of 25 per 10,000, while maternity bed density had a threshold of 10 per 1,000. After rescaling these values [i.e., minimum (100, X/threshold ^*^ 100)], where X is the bed density, they were combined into a hospital access composite variable for entry into the index calculations, computed as (Inpatient bed density ^*^ Maternity bed density)^1/2^. The thresholds chosen were based on the Malawi health facility assessment benchmark.

### Financial Risk Protection

As discussed earlier, two indicators were used to estimate the level of FRP: incidence of catastrophic healthcare expenditures and impoverishment due to OOP payments. The difference between CHEs and impoverishing health expenditures is that the latter can push a household into poverty or further into poverty if already below the poverty line. Table 4 summarizes the definition of the FRP indicators.

**Table 4:** Definitions of the financial risk protection indicators.

**Indicators**	**Numerator**	**Denominator**	**Data source**
Percentage of the population with no catastrophic healthcare expenditure	Number of households in the survey with no OOP spending exceeding 40% of their annual survey in the preceding 12-month period	Number of households in the survey	IHS4^a^
Percentage of households that did not get poor or pushed deeper into poverty by OOP spending	Number of non-poor households that did not get poor plus the number of poor households with no OOP spending	Number of households in the survey	IHS4

*OOP, out of pocket*.

a *IHS4 was used instead of IHS5 because the aggregated estimates to calculate the expenditure and poverty levels in IHS5 were not publicly available yet*.

### Data Sources

The following variety of data sources were used for the analysis: the 2015–2016 Demographic and Health Survey (DHS) (20) and the 2013–2014 Multiple Indicator Cluster Survey (MICS) (21) to analyze utilization and access of maternal and child health services; 2018 Facility Assessment Survey (11) used to analyze facility-level capacity; the MoH tuberculosis data extracted from the Ministry dataset used to analyze TB effective treatment; the MoH HIV/AIDS data extracted from the Ministry dataset to analyze access to ART for people living with HIV/AIDS; the Malawi STEP Survey (22) used to analyze non-communicable disease prevention and treatment services; and the 2016/2017 Integrated Household Survey (IHS) (23) used to analyze household-level expenditure on healthcare.

Both the DHS and MICS are household-based surveys collected by the Malawi National Statistics Office with technical assistance by the ICF through the DHS program. The surveys are conducted every 5 years and gather data on marriage, fertility, family planning, and reproductive and child health. The response rate for both surveys was very high, over 90%. The DHS program has policies in place of editing and imputation to ensure that the data accurately reflect the population studied, including sampling weights, median calculations, missing values, wealth index, and other tools of demography.

The 2018/2019 Malawi Harmonized Health Facility Assessment (HHFA) survey is a facility-level census that collects data on how equipped facilities are to provide primary healthcare in Malawi. This survey was conducted by the MoH with financial support from the Global Fund. The Malawi HHFA underwent an external quality assurance process by an independent team engaged by the Global Fund (11).

The Malawi IHS is also a household-based survey that collects comprehensive data on household expenditures, including spending on health, household, and individual demographic characteristics, outpatient care in the past 4 weeks prior to the survey, and inpatient care in the last 12 months prior to the survey. Both the inpatient and outpatient care data are collected at the individual level. The IHS is also collected by the National Statistics Office with technical assistance from the WB. To ensure data quality, sampling weights were used. Similar to the DHS and MICS, the response rate was very high at 93%.

Finally, the WHO STEPwise approach to NCD risk factor surveillance (STEPS) survey estimates the prevalence of non-communicable diseases (NCDs) and their risk factors in Malawi. The survey uses a standardized questions and protocols to allow comparisons across countries.

## Results

### Level of Universal Health Coverage in Malawi—UHC Index

The analysis showed that the inequality-adjusted UHC index (which was calculated as a geometric mean of the financial risk indicators and service coverage indicators) for Malawi is 69.68%, whereas the unadjusted UHC index is 75.03%. Further sensitivity analysis of the UHC index based on different weights and exclusion of service capacity indicators resulted in the following: 75.03% for unadjusted, 67.66% when both treatment and preventive indicators were given the same weight of 50%, and 61.83% when we included capacity indicators and maintained equal weights all throughout.

### Health Service Population Coverage

The findings of the key healthcare interventions at the population level are presented in Table 5. The utilization of ANC and family planning services remains very low in Malawi. Only 51% of women reported to having at least four ANC visits in 2016. The modern contraceptive prevalence rate is reported at 58%, whereas the met need for family planning services among married women is 59%. About 89% of women in Malawi received iron–folic acid supplements and at least two doses of TT injection during pregnancy, whereas 90 and 92% of the women reported to having been assisted by skilled personnel (doctor/nurse/midwife) and having delivered in a health facility, respectively.

**Table 5:** Coverage of health services in Malawi, 2015–2019.

**Indicators**	**Mean coverage** **(95% CI)**	**Concentration index (SE)**	**Inequality-adjusted estimates**
**Prevention indicators**			
Four or more ANC visits	50.77 (49.93–51.62)	0.07663616	43.11
Met need for family planning	59.22 (58.46–59.98)	0.05955617	53.27
Family planning prevalence	58.06 (57.28–58.82)	0.05359306	52.69
At least two tetanus injections	89.28 (88.76–89.81)	0.00924494	88.36
Folic and iron supplements	89.36 (88.84–89.88)	0.1419105	75.14
Full immunization	75.66 (74.18–77.14)	0.06168107	69.48
Bed net use by children under 5	43.46 (42.69–44.23)	0.14618039	28.85
Bed net use by pregnant women	46.95 (43.88–50.01)	0.16581048	30.39
Improved source of drinking water	87.13 (86.73–87.54)	0.29130189	58.01
Improved non-shared toilet facilities	51.76 (51.15–52.36)	0.17802085	33.96
Cancer screening	11.96 (10.73–13.18)	0.06166827	5.79
Non-smoking	75.50^a^	0.05620493	71.88
Geometric mean of prevention indicators	55.74	43.02	
**Treatment indicators**			
Baby postnatal care	63.02 (59.59–66.45)	0.04901072	58.15
Mother postnatal care	43.52 (42.65–44.40)	0.06182999	37.34
ARI treatment	73.44 (71.28–75.60)	−0.04992498	78.44
ORS treatment for diarrhea	64.74 (63.13–66.34)	0.01798706	62.93
Fever treatment	67.10 (65.77–68.45)	−0.02545055	69.64
Skilled birth attendant	90.31 (89.88–90.88)	0.17328123	73.06
Institutional delivery	92.27 (91.82–92.72)	0.21115801	71.15
Tuberculosis effective treatment	87.20	–	87.20
HIV treatment	80,70	–	80.70
Cardiovascular disease treatment	23.42 (19.06–27.78)	0.12034723	11.46
Management of diabetes	47.93 (30.51–65.34)	0.08129232	40.44
International Health Regulations core capacity index	56.13	–	56.13
Health professionals per capita (with threshold): physicians, psychiatrists, and surgeons	45.3	–	45.3
Hospital beds per capita (with threshold)	59.1	–	59.1
Proportion of health facilities with WHO-recommended core list of essential medicines available	38	–	38
Geometric mean of treatment indicators	58.66	55.77	
Overall service coverage index	57.77	51.59	

*95% CI, 95% confidence interval; ANC, antenatal care; ARI, acute respiratory infection; ORS, oral rehydration salts*.

a The actual figure was 88.75% before rescaling. Tobacco non-smoking is based on age-standardized estimates; as such, it is recommended that it is rescaled to provide finer resolution based on a minimum bound of 50%, so that the rescaled indicator = (X – 50)/(100 – 50) ^*^ 100, where X is the prevalence of tobacco non-smoking.

Although 76% of children in Malawi were fully immunized, mosquito net use was reported in less than half (50%) of children under 5 and pregnant women. About 87% of the households reported having access to clean drinking water, whereas only 52% reported having access to improved non-shared toilet facilities. Cervical cancer screening is significantly very low in Malawi, with only 11% of women reporting to having been tested for cervical cancer before. Similarly, for NCDs, the treatment rates are very low, with only 24 and 47% of the population being treated for cardiovascular disease (CVD) and diabetes, respectively.

The wealthy population had higher utilization rates for both preventive and treatment services in comparison to the poor, except for treatments for ARIs and fever, which mostly covered the poor (Table 5). The inequalities were much more evident for services such as iron–folic acid supplementation during pregnancy [concentration index (CI) = 0.142], utilization of ITN among the under 5 (CI = 0.146) and pregnant women (CI = 0.166), access to improved source of drinking water (CI = 0.291), access to improved non-shared toilet facilities (CI = 0.178), skilled birth attendance (CI = 0.173), institutional delivery (CI = 0.211), and CVD treatment (CI = 0.120). So much that, for all indicators, coverage was less after adjusting for inequality. The weighted summary index of SC was estimated to be 51.74%, whereas the unweighted SC was 53.99%.

### District-Level Estimates of the Service Coverage Index

**Figure 1:**
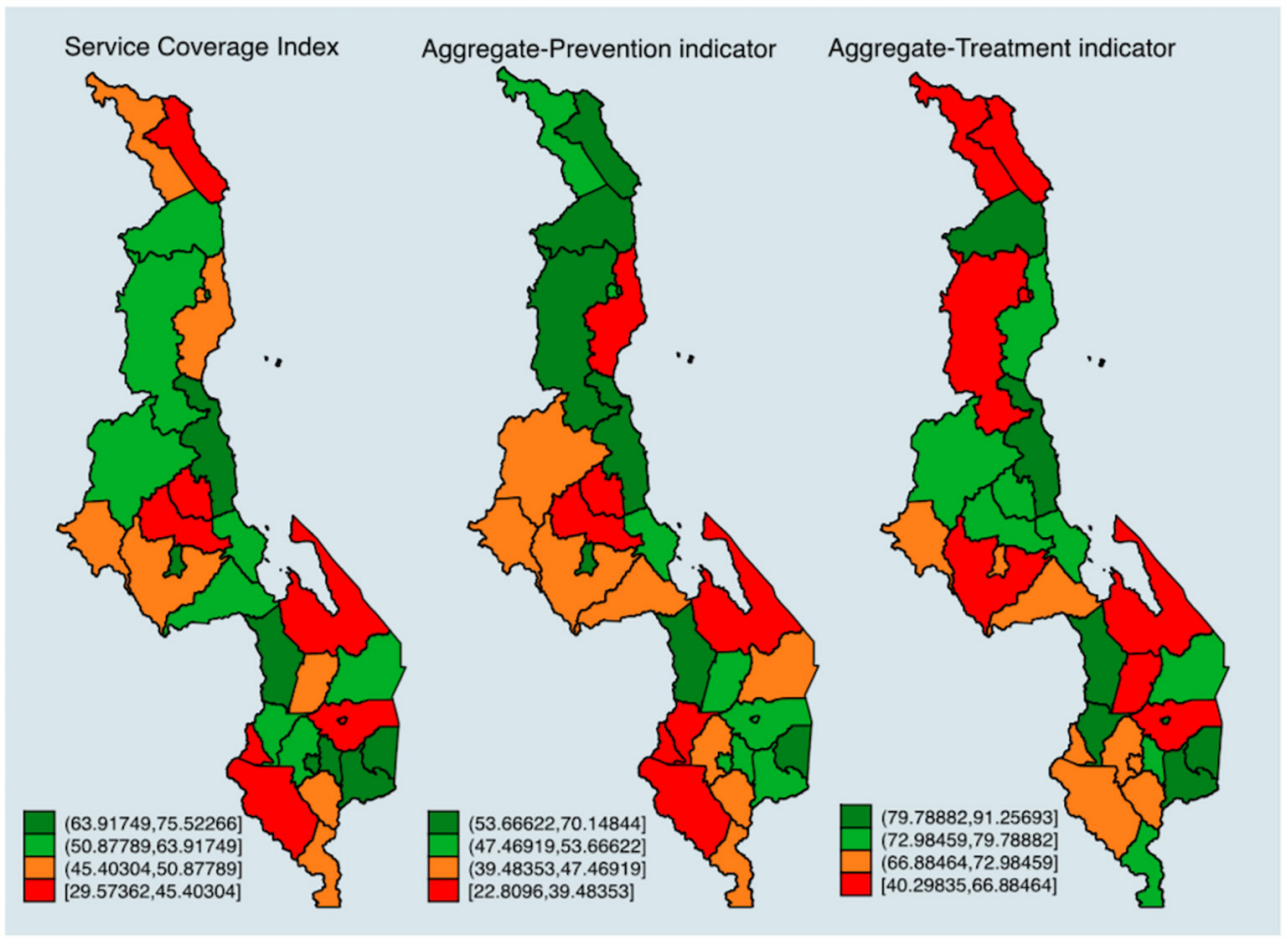
District level distribution of service coverage index. Authors based on the 2015/2016 DHS data.

Figure 1 shows the district-level coverage of selected SC indicators from the DHS without the service capacity indicators included. The darker the shade of green, the better the service coverage. The district-level estimates showed geographic variations in the population coverage of services, with the Southern and the Central regions having high service coverage compared to the Northern region.

### Financial Risk Protection

The findings for FRP are provided in Table 6 and showed that the percentage of households with catastrophic healthcare spending is estimated at 1.3%. The results further showed that the poor are more likely to incur CHEs. Additionally, about 1.42% of households got poor due to healthcare expenditures. Furthermore, among the poor households, over 2% were pushed further into poverty due to healthcare expenditures, giving a total of 3.75% of households being impoverished due to catastrophic healthcare expenditures. These estimates gave summary measures of FRP of 97.45% before adjusting for inequality and 94.10% after inequality adjustment.

**Table 6:** Financial risk protection in Malawi.

**Indicators**	**Mean coverage** **(95% CI)**	**Unadjusted FRP index**	**Concentration index (SE)**	**Inequality-adjusted index**
**Financial risk indicators**				
Incidence of catastrophic expenditures (*a*)	1.33835% (1.14–1.54)		−0.0665735	1.339526489
Percentage of the population that got poor (*b*)	1.42		–	–
Percentage of the population pushed deeper into poverty (*c*)	2.33		–	–
Impoverished indicator (*b* + *c*)	3.75%		–	–
**Final financial risk indicators**				
Percentage of households with no catastrophic health expenditures (100 – *a*)	98.66 (98.46–98.86)	98.66%	0.0665735	92%
Percentage of households that were not impoverished by OOP spending: 1 – (*b* + c)	96.25	96.25%	–	96.25%
**Summary measure of FRP**				
Financial protection index	97.45		94.10	

*FRP, financial risk protection*.

## Discussion

The commitment to UHC both at the national and global levels has emphasized the importance of measuring it to track its progress over time (1, 2). In this paper, we have presented an analysis of Malawi's current status on UHC to provide a basis on which to monitor UHC progress moving forward. For the analysis, we adopted the WHO/WB UHC monitoring framework (9) and suggestions by Wagstaff et al. (2) and Barasa et al. (7).

There are a number of observations that we made. Firstly, looking into the individual UHC dimensions, the SC level was found to be low at 51.59%. This implies that slightly over half of the population have access to both preventive and treatment service indicators. Given that the SC index coverage ranges between 0 and 100%, with high values signifying better coverage, 51.59% is relatively very low and implies that service coverage is inadequate and that, in order to achieve SDG goal 3.81, there is still more that needs to be done. Although the SC level is low, it is significantly higher than the estimate reported by the WHO for Malawi of 44% in 2015 (9), probably because the most recent data they used were for 2010, especially on TB treatment effectiveness and HIV treatment. For this analysis, the HIV and TB data were taken from the MoH, which has relatively higher and the most recent estimates. Nevertheless, the low SC agrees with findings from Hogan et al. (5), which showed low SC indices for LMICs in Sub-Saharan Africa (42%) and southern Asia [53]. The findings are also similar to those reported by Barasa et al. (7) in Kenya who found a low SC of 42% in 2013.

With regard to the service coverage domains, for the prevention indicators, Malawi is doing well in the following areas: tetanus injection, access to improved water source, folic and iron supplements during pregnancy, and full immunization, with estimates ranging between 77 and 89%. However, although maternal and child health services are provided as part of the essential healthcare package and therefore provided for free in all public facilities and selected CHAM facilities (16), ANC and family planning coverage still remains relatively low and pro-rich. The proportion of women reporting to have at least 4 ANC visits is only at 50%, and the family planning prevalence rate is only 58%. Although the lower ANC utilization rate is puzzling, there are possible reasons that could explain this phenomenon. For instance, results for a study in rural Mangochi reported that the focused antenatal care (FANC) requirement for birth preparedness which demanded that pregnant women bring a baby wrapper (traditional “chitenje”) at the first FANC visit was costly to most rural poor women and made them shun away from using FANC services (24). There is a need for more community advocacy educating women on the importance of ANC in pregnancy. Given that there are community health workers in most rural areas of Malawi, the government should support them to reach out to women and men in their communities.

Similarly, the use of insecticide-treated mosquito nets is generally low among pregnant women and children under 5 despite the nationwide mass ITN distribution campaigns that have been taking place since 2012, with the most recent one happening in 2018 (25). The low ITN use is a concern in the fight to eliminate malaria by 2030, given that Malawi is among the countries with high malaria prevalence rates. The expansion of access to priority healthcare services to the population is a priority area for the Malawi HSSP II (10). The goal is that all the people of Malawi should lead a quality and productive life (10). On the other hand, regarding the treatment indicators, the high rates of skilled and institutional deliveries illustrated the impact of government policies to ensure safe deliveries for both mother and baby.

Secondly, although most of the government policies mainly target poor households, substantial inequalities in healthcare access between the rich and the poor and between rural and urban still exist. This finding is in agreement with the findings reported in the 2017 WHO/WB Global Monitoring Report, which found that only 17% of the poorest households in LMICs received at least six basic health interventions in comparison to 74% of households in the richest quintile (9). This highlights the argument that, without deliberate and proactive efforts to ensure equity, policy reforms aimed at achieving UHC may favorably benefit the rich and exclude the poor, resulting in health systems that are inequitable (26). Given that equity is at the core of UHC in healthcare (9), it is therefore important that progress is made equitably (27, 28).

With regard to FRP, we found that 94.10% of the individuals in our sample were financially risk protected. Our estimate was much higher than that reported in Kenya at 63.78% in 2013 (7). The following are the possible reasons for this high figure in Malawi. Firstly, primary healthcare in Malawi is provided for free in all government facilities, whereas in areas with no public facilities, since 2006, the government entered into an agreement with CHAM^1^ facilities to remove user fees for selected primary healthcare services. Evidence showed that the agreement between the government of Malawi and CHAM facilities led to reduced OOP expenditures by the poor and also to increased coverage and utilization of maternal and child health services (16). Although the high FRP indicator implies that Malawi is doing well on SDG goal 3.8.2, the poor bear the largest burden of catastrophic healthcare costs. This is consistent with findings that OOP is typically regressive and stresses the need to implement progressive prepayment mechanisms (7, 29–31).

Furthermore, the percentage of Malawians pushed deeper into poverty is higher than the percentage of households who got poor due to CHEs. This means that any OOP made by poor households toward healthcare services makes them worse off. This underscores the need for Malawi to still prioritize not only service coverage but also FRP. The major challenge that impedes the provision of quality health services for all in Malawi is that healthcare financing remains unsustainable and unpredictable (11). As shown in Table 1, donors are the main financiers of total health expenditure (THE), with contributions of 57.6%; at 24.4%, the government of Malawi is the second main funder of THE. Household contribution toward THE is estimated at 12.7%. Although the overall healthcare resource envelope increases with donor funds, the majority of the funds are allocated for vertical programs, such as HIV/AIDS to which donors contribute 95% of the total financing (19). Since funds for vertical programs are earmarked for specific programs, they therefore cannot be reallocated to other priority areas and hence distort the healthcare priorities and lead to health system inefficiencies (9). Additionally, due to fragmentation of the donor funds and lack of on-budget support, planning is increasingly difficult (14).

Currently, Malawi faces a lot of challenges to effectively implement a social health insurance scheme, among them is a narrow formal sector from which to collect premiums (32), which means that over-reliance on OOP will still remain, exposing households to catastrophic payments and impoverishment and preventing progress toward UHC. Another challenge is that healthcare purchasing in Malawi is passive and not strategic, which compromises quality, equity, and efficiency (14).

The overall UHC index was computed by estimating the geometric mean of the summary measure of the service coverage and FRP indicators. The UHC index values ranged between 0 and100%, with values closer to 100 and close to 0 implying high UHC and low UHC, respectively. We found that the overall UHC index for Malawi is 69.68%, which, compared to estimates found in other LMICs, is significantly higher. For instance, Barasa et al. (7) found a UHC index of 52% in Kenya, Prinja et al. (8) found a UHC index of 53% in India, and Wagstaff et al. (2) reported a UHC coverage ranging between 51 and 57% for 1998–2006 in selected low-income countries using the same methods.

Nevertheless, the high UHC index in Malawi can be explained as follows: firstly, although the coverage for most services is high, some service coverage indicators still remain pro-rich; this non-use by the poor is shown in the low levels of OOP payments and the high FRP score (94.10%), and hence a relatively higher UHC score. This finding is similar to the results reported by Wagstaff et al. (2) in Ethiopia, where the service coverage summary measure was low at 35%, but the country had a very high FRP summary measure of over 90%. Secondly, although primary healthcare is free at the point of service, the quality of care is very low, with regular drug stockouts and low client satisfaction and distrust in the provision of public health services (26). These factors may lead to increased OOP expenditures as people may be forced to seek alternative sources of care, i.e., from private facilities. However, given that the majority of the population is poor, they would rather forgo healthcare access than risk the financial burden of accessing care in private facilities or buying medicines at the pharmacy; this is reflected in the high FRP estimate.

### Study Policy Implications and Recommendations

Drawing from our findings that service coverage of essential healthcare services is very low at 51.59% and the evidence available from other public documents, for instance the National Health Accounts (14), we made several recommendations that could put Malawi on the path to attaining UHC by 2030. Firstly, to ensure adequate financing in the health sector, the government should focus on reducing the inefficiencies and increasing domestic resource mobilization. An increase in domestic resources would ensure sustainability in health financing and could likely reduce challenges such as consistent drug stockouts. Consistent drug stockouts in public facilities is one of the factors that force households to buy over-the-counter medication or go to private facilities using OOP resources, hence exposing them to the risk of incurring CHEs (10).

Secondly, to ensure quality, efficiency, and equity in the provision of healthcare services in Malawi, there is a need to embrace strategic^2^ purchasing practices. For example, currently, the financing and payment for EHP and non-EHP interventions are not clearly separated (14). For EHP interventions to be delivered effectively, the payment and financing of EHP and non-EHP interventions need to be separated through a functional system. Improvements in the delivery of EHP services could potentially increase their uptake and, hence, service coverage.

In addition, there is vast empirical evidence that primary healthcare is the cost-effective delivery route to attaining UHC; as such, Malawi should consider prioritizing and strengthening primary and community healthcare facilities and systems. Finally, currently, Malawi does not have social health insurance, which explains why there are poor people who are pushed deeper into poverty due to OOP expenditures. The government should consider the implementation of a social insurance scheme to provide full protection from OOP expenditures.

### Strengths and Limitations

The major strength of the study is its incorporation of indicators that captured the UHC core dimensions and the supply and demand side factors, which, in comparison to other studies available in Malawi, presented a more comprehensive perspective of the linkages of socioeconomic inequalities in health and healthcare.

Nevertheless, we would like to acknowledge the following important study limitations. Firstly, due to data limitations, our analysis did not include neglected tropical diseases such as bilharzia and trachoma, among others, which are mostly prevalent in rural areas; as such, our results may have overestimated the level of UHC. The development of the UHC index was entirely dependent on data availability, which means that the analysis is not a perfect representation of the full dynamics of the access pathway. Secondly, the dependence on self-reported data is another limitation given that such data depend on respondents' intellectual ability and socio-demographic characteristics (33). For example, a better assessment of the healthcare needs by the relatively wealthy may be a function of their better education outcomes. Besides, self-reported data are also prone to recall bias/errors due to longer recall periods. For example, the household surveys asked about the services received 5 years prior to the survey. We tried to reduce recall errors by limiting the sample in the DHS to women who reported to have given birth 2 years prior to the survey and that the baby was still alive.

Thirdly, the household surveys only included data from respondents who agreed to be interviewed; as such, there may be sample selection bias. As a result, the results were not a full representation of the situation in Malawi, but rather indicative. Lastly, as with most household surveys, there were missing variables due to non-responsiveness or error in data entry, which could potentially lead to an over/underestimation of UHC.

In terms of areas of future research, we would suggest the development of district level UHC index to measure the progress of UHC at the district level. This would be critical to identify specific areas that each district needs to work on and provide tailored interventions for the districts to improve their figures for UHC.

## Conclusion

This study developed a composite UHC index for Malawi using the most recent data available. Our findings showed that, while Malawi is doing moderately well on the overall UHC level, service coverage is still low and inequalities in both UHC dimensions remain a concern. A commitment to attaining UHC by 2030 means that evidence-based health financing and other health sector reforms should be made to achieve this goal. As evidenced from the results of the study, such reforms should be a combination of both UHC dimensions and not only either of the dimensions.

## Data Availability Statement

Publicly available datasets were analyzed in this study. This data can be found here: https://dhsprogram.com/data/available-datasets.cfm; https://microdata.worldbank.org/index.php/catalog/3818; https://extranet.who.int/ncdsmicrodata/index.php/catalog/629/get_microdata.

## Author Contributions

MM conducted the data analysis and drafted the manuscript. GM conceived the study and reviewed the manuscript. AC and EC reviewed the manuscript. All authors contributed to the article and approved the submitted version.

## Conflict of Interest

The authors declare that the research was conducted in the absence of any commercial or financial relationships that could be construed as a potential conflict of interest.

## Publisher's Note

All claims expressed in this article are solely those of the authors and do not necessarily represent those of their affiliated organizations, or those of the publisher, the editors and the reviewers. Any product that may be evaluated in this article, or claim that may be made by its manufacturer, is not guaranteed or endorsed by the publisher.

## Footnotes

^1^CHAM facilities provide 75% of the primary healthcare services in rural and hard-to-reach areas, where public facilities are not available.

^2^Strategic purchasing is when decisions about what to buy (benefit package), from whom to buy, and the buying mechanisms from the providers are structured such that they optimize the health system goals of equity, efficiency, and quality (14).
